# Antimicrobial resistance in *Escherichia coli* from Swedish piglets with diarrhoea and associations with potential risk factors

**DOI:** 10.1186/s13028-025-00795-9

**Published:** 2025-04-02

**Authors:** Estelle Ågren, Annette Backhans, Maria Lindberg, Marie Sjölund, Björn Bengtsson, Arianna Comin

**Affiliations:** 1 Department of Epidemiology and Disease Control, Swedish Veterinary Agency, Uppsala, SE-751 89 Sweden; 2 Department of Animal Health and Antimicrobial Strategies, Swedish Veterinary Agency, Uppsala, Sweden; 3 Farm and Animal Health, Kungsängens Gård, Uppsala, SE-753 23 Sweden; 4 Department of Clinical Sciences, Swedish University of Agricultural Sciences, Uppsala, SE-750 07 Sweden

**Keywords:** Antibiotic/antimicrobial resistance, *Escherichia coli*, Neonatal diarrhoea, Pig/swine/porcine, Post-weaning diarrhoea, Risk factor

## Abstract

**Background:**

Antibiotic treatments of diarrhoea in suckling piglets and in pigs after weaning are common worldwide and contribute to antimicrobial resistance (AMR) in *Escherichia coli* from pigs. In Sweden, during the last decades, resistance to trimethoprim-sulphonamide and ampicillin has increased markedly in *E. coli* from routine clinical samples from piglets with diarrhoea, hereafter referred to as “clinical submissions”. This has occurred despite a comparatively low use of antibiotics in Swedish pig production. However, clinical submissions might be biased towards farms with treatment failures and therefore overestimate occurrence of AMR. To explore the representativeness of data from such samples we compared occurrence of AMR in *E. coli* from clinical submissions and from concurrent samples collected from piglets with diarrhoea by convenience, referred to as “study samples”. We also investigated associations between farm-related potential risk factors and AMR using farm data collected through a questionnaire. Data were evaluated using univariable and multivariable statistical models, as well as a multivariate model.

**Results:**

In all, 158 study samples from 97 herds and questionnaires from 83 herds were analysed. Resistance to streptomycin (37%), trimethoprim-sulphonamide (32%), ampicillin (30%), and tetracycline (18%) were the most frequent traits. Occurrence of AMR in 158 *E. coli* isolates from study samples was not significantly different from occurrence in 57 isolates from concurrent clinical submissions (*P* > 0.05). In 70% of herds, more than 10% of the sows were treated with antibiotics in the first week after farrowing, and trimethoprim-sulphonamide was the most common first choice antibiotic. Trimethoprim-sulphonamide resistance was associated with the proportion of sows receiving post-farrowing treatment. Resistance to ampicillin, tetracycline, and streptomycin resistances were indirectly associated with sow treatments, likely via co-resistance to trimethoprim-sulphonamide. There was no significant association between high dose zinc oxide supplementation and AMR (*P* > 0.05).

**Conclusions:**

Clinical submissions do not overestimate occurrence of AMR in *E. coli* from Swedish piglets with diarrhoea and are therefore relevant for AMR monitoring. Even at low treatment rates, post-farrowing treatment of sows increases the risk for AMR in piglets. This applies especially for trimethoprim-sulphonamide resistance, but also for resistance to other antibiotics, and indicates that antibiotic use must be reduced substantially to achieve a reduction of AMR.

## Background

Antimicrobial resistance (AMR) is an increasing threat throughout the world and linked to the use of antibiotics [1]. In pigs, antibiotic treatment is common in both suckling piglets with neonatal diarrhoea and in pigs after weaning with post-weaning diarrhoea [2]. This contributes to antibiotics use in pigs worldwide and thereby emergence of AMR among *Escherichia coli* in pigs [3].

Data from the Swedish antimicrobial resistance monitoring program (Svarm) show that both trimethoprim-sulphonamide and ampicillin resistance in clinical *E. coli* isolates from pigs increased markedly in the last two decades, from a three-year moving average around 10% in the late 1990s to around 35% in the late 2010s [4]. This has occurred although sales of antibiotics for food producing animals in Sweden are among the lowest in Europe [5], and most treatments in pigs are therapeutic treatments of individual animals [6]. Therefore, this increase raised concerns that the samples used to monitor AMR might be biased. In Svarm the monitoring of *E. coli* from piglets with diarrhoea is based on clinical samples submitted by veterinary practitioners to the routine bacteriologic laboratory at the National Veterinary Agency (SVA). These samples are hereafter referred to as “clinical submissions”. During the last years there has been a successive decrease in the number of clinical submissions to SVA which could, in theory, be due to a shift towards cases with treatment failures. If so, occurrence of AMR in *E*. *coli* from piglets with diarrhoea could be overestimated in Svarm. Furthermore, a national regulation from 2013 [7] require susceptibility testing before prescription of quinolones to food producing animals. This could also have added to a selection of submissions from herds where therapeutic failures with the recommended first line antibiotic trimethoprim-sulphonamide have been noted.

Another concern was whether the past extensive use of zinc oxide in Swedish pig herds to prevent post-weaning diarrhoea could be an explanation for the high occurrence of resistance in *E. coli*. Zinc oxide is used in approximately 40% of Swedish herds [8] and it has been reported that zinc oxide could select for AMR in *E*. *coli* [9, 10].

The first aim of this study was to explore if AMR levels for *E. coli* from piglets with diarrhoea, based on clinical submissions, in Svarm are representative. A second aim was to look for potential causes for the high occurrence of AMR in *E. coli* from piglets with diarrhoea.

## Methods

In this observational epidemiologic study, faecal swab samples from piglets with diarrhoea were collected by farmers. These samples are hereafter referred to as “study samples” and were collected without an intent to clarify clinical problems in a herd. They were therefore free from the possible biases of ordinary clinical submissions. Study samples were accompanied by herd data obtained via a questionnaire during a face-to-face interview when a farm was enrolled in the study. *Escherichia coli* cultured from the study samples were tested for antimicrobial susceptibility and occurrence of AMR was compared to occurrence of AMR in *E. coli* from concurrent clinical submissions to SVA. In addition, associations between farm-related potential risk factors, such as treatments with antibiotics or zinc oxide, and occurrence of AMR were investigated. Results were described and checked for associations by regression analyses and Additive Bayesian Network (ABN) modelling.

### Enrolment of herds and collection of study samples

Veterinarians employed by the Swedish Farm & Animal Health veterinary advisory organisation were asked to enrol the next ten herds in which they were going to perform a routine visit. At the visit, the farmer was invited to participate in the study. When a farmer declined to participate, the farmer on the next routine herd visit was invited instead. Enrolled farmers were asked to collect faecal swab samples from piglets with diarrhoea on the next occasion of diarrhoea in the herd; one sample from a suckling piglet (≤ 1 week old and not treated with antibiotics) and one sample from a weaned piglet (≤ 3 weeks after weaning, not treated with antibiotics). Samples were collected using cotton swabs (Amies charcoal media, Copan Diagnostics, Brescia, Italy) and sent by mail to SVA for analysis. The samples were accompanied by information on age category (suckling piglet/weaned piglet), if the sow to the sampled suckling piglet had received antibiotic treatment during the week prior to sampling (yes/no), and type of treatment if applicable. Samples were collected from June 2016 until June 2017.

### Questionnaire on herd information

During the routine visit when the farmers were enrolled, they were also asked to answer five multiple choice questions and one open question in a questionnaire. The questions were about type of feed given at weaning, use of high dose zinc oxide supplementation, i.e. supplementation of 2500 ppm zinc oxide to feed for the first two weeks after weaning, first-choice antibiotic for neonatal and post-weaning diarrhoea, estimation of the proportion of sows treated with antibiotics within the first week post-farrowing (open question), and a question on the first-choice antibiotic for post-farrowing treatments (Table 1). The questionnaire was answered together with the veterinarian and thereafter sent to SVA. The questionnaires and the study samples were coded and thereby linked anonymously.

**Table 1:** Questionnaire responses regarding risk factors for antimicrobial resistance from 83 pig farmers

Question	Response alternatives	Number of answers	Proportion of answers (%)
Type of feed given at weaning?	Liquid feed	20	0.24
	Dry feed	22	0.27
	Both liquid and dry feed	41	0.49
Supplementation of high dose zinc oxide to the feed?	Ongoing	34	0.41
	During the past year	2	0.02
	More than one year ago	16	0.19
	Never	31	0.37
First choice antibiotic for neonatal diarrhoea?	Trimethoprim-sulphonamide	48	0.58
	Ampicillin ^a^	10	0.12
	Penicillin	4	0.05
	Enrofloxacin	1	0.01
	Other ^b^	20	0.24
First choice antibiotic for post-weaning diarrhoea?	Trimethoprim-sulphonamide	61	0.73
	Ampicillin	0	0.00
	Colistin	4	0.05
	Neomycin	1	0.01
	Enrofloxacin	0	0.00
	Other ^c^	17	0.20
Estimated proportion of sows receiving antibiotic treatment within the first week after farrowing?	< 10%	25	0.30
	10–19%	37	0.45
	20–29%	16	0.19
	≥ 30%	5	0.06
First choice antibiotic for post-farrowing treatment?	Trimethoprim-sulphonamide	58	0.70
	Ampicillin	0	0.00
	Penicillin	17	0.20
	Enrofloxacin	0	0.00
	Other ^d^	8	0.10

^a^ Amoxicillin (*n* = 6) and ampicillin (*n* = 4) were combined in the ampicillin category; ^b^ tetracycline/doxycycline (*n* = 3), florfenicol (*n* = 1), colistin (*n* = 2), neomycin (*n* = 5), trimethoprim-sulphonamide and ampicillin/amoxicillin (*n* = 2), trimethoprim-sulphonamide and penicillin (*n* = 1), trimethoprim-sulphonamide and neomycin (*n* = 5), trimethoprim-sulphonamide and neomycin (*n* = 1); ^c^ tylosin (*n* = 14), trimethoprim-sulphonamide and colistin (*n* = 2), trimethoprim-sulphonamide and tylosin (*n* = 1); ^d^ trimethoprim-sulphonamide and penicillin (*n* = 7), penicillin and dihydrostreptomycin (*n* = 1)

### Bacteriology and susceptibility testing of study samples

All analyses were performed at SVA. Study samples were cultured on agar plates with 5% horse blood (SVA, Uppsala, Sweden) and on plates with bromocresol purple–lactose agar (SVA, Uppsala, Sweden) following routines for enterotoxigenic *E. coli* (ETEC) diagnostics as previously described [11]. It was noted if colonies causing haemolysis were present and one colony per plate, haemolytic if present, was selected, confirmed as *E. coli* using MALDI-TOF MS (MALDI Biotyper system, Bruker Daltonics, Bremen, Germany) and tested by polymerase chain reaction (PCR) for the enterotoxin genes LT, Sta [12, 13] and STb [14]. *Escherichia coli* with enterotoxin genes (ETEC) were further tested for the fimbrial adhesins F4, F5, F18, F41 and F6 by PCR [14]. Samples were also screened for quinolone resistant *E. coli* (QREC) by selective culture on MacConkey agar supplemented with nalidixic acid at 32 mg/L (SVA, Uppsala, Sweden).

Antimicrobial susceptibility testing was performed using microdilution panels (VetMIC, SVA, Uppsala, Sweden) and minimum inhibitory concentration (MIC) in mg/L was read as the lowest concentration for each substance that inhibited bacterial growth. MICs were interpreted by EUCAST (European committee on antimicrobial susceptibility testing) epidemiological cut-off values for resistance i.e.: ampicillin > 8 mg/L, cefotaxime > 0.25 mg/L, colistin > 2 mg/L, enrofloxacin > 0.12 mg/L, gentamicin > 2 mg/L, neomycin > 8 mg/L, nitrofurantoin > 64 mg/L, streptomycin > 16 mg/L, tetracycline > 8 mg/L, trimethoprim-sulfamethoxazole > 1 mg/L.

The analyses provided the following variables: nalidixic acid resistance (yes/no) for the samples and for the isolates; haemolysis (yes/no), enterotoxin genes (yes/no), and antimicrobial resistance to ten types of antibiotics (yes/no).

### Data on isolates from clinical submissions

For comparison, results for susceptibility tested *E. coli* from clinical submissions of pig samples to SVA were retrieved from the laboratory data system. These samples were clinical samples submitted by practitioners, either as faecal swabs collected from live pigs with diarrhoea or samples from the gastrointestinal tract collected at post-mortem examinations, during June 2016 until June 2017 i.e., the same period as the collection of samples within the study. Samples of clinical submissions were analysed using the same laboratory methods as study samples, but for most samples two colonies were picked from primary cultivation and further tested by PCR. Also, in most cases, only ETEC positive isolates were susceptibility tested.

### Data management

All data from questionnaires were manually entered into an Excel spread sheet and combined with laboratory data on samples and isolates.

Concerning variables from the submission form accompanying study samples and from the questionnaire, amoxicillin, and ampicillin, that belong to the same class of antibiotics, were combined into one category named ampicillin. Likewise, doxycycline and tetracycline were combined to one category named tetracycline. For high dose zinc oxide supplementation, the categories “ongoing” and “during the past year” were combined into one category, due to a small number of herds (*n* = 2) in the latter category. Estimations on proportion of sows receiving post-farrowing treatment clustered on some values, therefore this variable was categorised into four categories (< 10%, 10–19%, 20–29% and ≥ 30%).

### Statistical analyses

Univariable analyses was used comparing AMR in study samples and clinical samples. Associations were tested with chi-squared tests or, if the number of observations in any category/outcome group was small (*n* < 10), with Fischer´s exact test. Associations with a *P*-value < 0.05 were considered significant.

Univariable analysis was also performed on study samples by generating contingency tables with all combinations of the variables, i.e., variables on herd information from the questionnaire, variables on sample information from the submission form and variables on properties of the *E. coli* isolates from laboratory tests. Associations were tested with chi-squared tests or, if the number of observations in any category/outcome group was small (*n* < 10), with Fischer´s exact test. Associations with a *P*-value < 0.05 were considered significant.

For multivariable analysis, hierarchical mixed logistic regression was used to investigate associations to trimethoprim-sulphonamide resistance. Trimethoprim-sulphonamide resistance was used as outcome, as this was the dominating first choice antibiotic for treatment of piglet diarrhoea as well as for post-farrowing treatments. Five fixed variables were tested, selected based on the results of the univariable analyses, these were: proportion of sows receiving post-farrowing treatment; trimethoprim-sulphonamide as first choice antibiotic treatment for neonatal diarrhoea, post-weaning diarrhoea or post-farrowing treatment of sows; and use of high dose zinc oxide in feed at weaning. Herd was included as a random variable to investigate and account for potential clustering of the outcome variable at herd level. Manual stepwise backward elimination was used. Explanatory variables with a *P*-value ≤ 0.05 were kept in the final model. Uni- and multivariable analyses were performed in Stata version 13 (StataCorp. 2013. Stata Statistical Software: Release 13. College Station, TX: StataCorp LP).

To improve understanding of the inter-relationships between all variables of interest, a multivariate analysis was performed by means of additive Bayesian network (ABN) modelling [15, 16]. Variables with all, or almost all, answers or analytical results in the same category were excluded, because they did not have enough variation to provide evidence of statistical association. The remaining variables that were included in the model were high dose zinc oxide supplementation in feed currently or during the past year (no/yes), if more than 10% of sows received post-farrowing antimicrobial treatment (yes/no), age category (suckling piglets/weaned piglets), nalidixic acid resistance, and properties of the isolate, namely; antimicrobial resistance to trimethoprim-sulphonamide, tetracycline, ampicillin, and streptomycin respectively (resistant / not resistant), presence of enterotoxin genes (yes/no), and haemolysis (yes/no).

ABN modelling is a modern graphical approach for structure discovery, suitable when looking for statistical dependencies between many inter-related variables in a dataset. The method attempts to determine an optimal statistical model (i.e., graphical structure) directly from observed data, allowing all variables to be potentially mutually dependent. It further allows discrimination between indirect and direct associations, by estimating the joint probability distribution of all variables of interest [15]. The modelling was performed at sample level, as the preliminary hierarchical logistic analysis as well as manual inspection of data showed no clustering of AMR results at herd level. The ABN models comprise of two reciprocally dependent parts: (i) the structure, outlined graphically by a directed acyclic graph (DAG) and (ii) the marginal probabilities, which can be seen as the analogous of regression coefficients. Both structure discovery and parameter learning are identified by Bayesian algorithms, which attempt to find the model most supported by the data. For the analysis in this study, uninformative parameter priors were used. The optimal model was identified by an exact search of the data [17] iterated across incremental parent limits (using the package ‘abn’ [18]), followed by parametric bootstrapping to adjust for potential overfitting of the model [19]. Bootstrapping included simulating 1000 new datasets from the initial ABN results, and only keeping the associations appearing in at least 50% of the resulting DAGs [16]. To stress that associations identified did not contain information on causality, the directionality of the arcs was removed in the DAG presented. The additive Bayesian network modelling was performed in R [20] and JAGS [21] and Graphviz [22] was used to visualize the DAG.

## Results

### Herd data

In all, 158 study samples, 86 from suckling piglets and 72 from weaned piglets. The samples were from 97 herds of which 61 sent in two samples, one from a suckling and one from a weaned piglet whereas 36 herds sent in only one sample either from suckling (25 samples) or weaned piglets (11 samples). Among the samples from suckling piglets, only five were from litters where the sow had received antibiotic treatment in the last week.

Questionnaires were available from 83 of the herds, and there were no missing answers in any of these questionnaires (Table 1). Fourteen herds did not send in questionnaires, but nine of these herds submitted samples from both suckling and weaned piglets whereas five herds submitted only one sample each, either from a suckling or from a weaned piglet.

Trimethoprim-sulphonamide dominated as first-choice for post-farrowing treatment of sows, as well as for neonatal and post-weaning diarrhoea (Table 1). In 21 of the 83 herds, the farmer estimated that more than 19% of the sows were treated with antibiotics in the first week after farrowing, in 37 herds 10–19% of the sows were treated, and in 25 herds less than 10% of the sows were treated (Table 1). High dose zinc oxide supplementation was used in 34 herds (41%) (Table 1).

### Bacteriology and antimicrobial susceptibility of study samples

The bacterial cultivation of study samples resulted in 158 isolates of *E. coli*, 86 from suckling and 72 from weaned piglets. None of the isolates from suckling piglets were haemolytic compared to 26 from weaned piglets (36%). Enterotoxin genes were found in 11 of the 86 isolates (13%) from suckling piglets and in 29 of the 72 isolates (40%) from weaned piglets. In isolates positive for enterotoxin genes, fimbrial adhesins were detected in four of the isolates from suckling and in 20 isolates from weaned piglets. Nalidixic acid resistant colonies were found in 33 samples (38%) from suckling and from 24 samples (34%) from weaned piglets.

Sixty-five of the 158 isolates (41%) from study samples were susceptible to all tested antibiotics. Resistance to at least one antibiotic was detected in 93 of the isolates (59%), with streptomycin resistance (37%), trimethoprim-sulphonamide resistance (32%), ampicillin resistance (30%), and tetracycline resistance (18%) detected most frequently (Table 2). Resistance to cefotaxime was not found and colistin resistance was found in three (2%) isolates. Resistance to more than one antibiotic was detected in 59 isolates (37%) and 31 isolates (20%) were resistant to three or more antibiotics and thus multiresistant (Table 3). Co-resistance to trimethoprim-sulphonamide, streptomycin and ampicillin, often in addition to other antibiotics, was the most common resistance phenotype and found in 39 isolates (25%), 10 of these isolates were resistant also to tetracycline. The proportion of sows receiving post-farrowing antibiotic treatments in relation to trimethoprim-sulphonamide resistance in isolates is shown in Table 4.

**Table 2:** Occurrence of antimicrobial resistance in *Escherichia coli* from study samples and from clinical submissions

**Antibiotic**	Occurrence of resistance (%)	
	**Study samples** (*n* = 158)	**Clinical submissions** (*n* = 57)
Ampicillin	30	37
Cefotaxim	0	0
Colistin	3	0
Enrofloxacin	8	9
Gentamicin	0	4
Neomycin	5	12
Nitrofurantoin	0	0
Streptomycin	37	40
Tetracycline	18	23
Trimethoprim-sulphonamide	32	35

Occurrence of antimicrobial resistance in isolates from study samples were compared with results from concurrent clinical submissions. No significant differences were observed (*P* > 0.05)

**Table 3:** Resistance phenotypes of *Escherichia coli* from study samples

Resistance phenotype ^a^							Number of isolates
Sm	Tmp-Sulfa	Am	Tc	Ef	Nm	Col	
R	R	R	R	R	-	-	3
R	R	R	R	-	R	-	1
R	R	R	R	-	-	-	6
R	R	R	-	R	-	-	1
R	R	R	-	-	R	-	2
R	R	R	-	-	-	R	1
R	R	-	R	-	R	-	1
-	R	R	R	-	R	-	1
R	R	R	-	-	-	-	14
R	R	-	R	-	-	-	1
R	R	-	-	-	R	-	1
R	-	R	R	-	-	-	4
-	R	R	R	-	-	-	1
-	R	R	-	R	-	-	2
R	R	-	-	-	-	-	8
R	-	R	-	-	-	-	1
R	-	-	R	-	-	-	2
R	-	-	-	R	-	-	1
-	R	R	-	-	-	-	4
-	R	-	-	-	R	-	1
-	-	R	R	-	-	-	1
-	-	R	-	-	-	R	1
-	-	-	R	-	-	R	1
R	-	-	-	-	-	-	12
-	R	-	-	-	-	-	2
-	-	R	-	-	-	-	5
-	-	-	R	-	-	-	7
-	-	-	-	R	-	-	6
-	-	-	-	-	R	-	1
-	-	-	-	-	-	R	1

Antimicrobial resistance phenotypes for the 59 isolates of *E. coli* from suckling and weaned piglets with diarrhoea that were resistant to at least one antibiotic. R indicates resistance. ^a^ Sm, streptomycin; Tmp-Sulfa, trimetoprim-sulphonamide; Am, ampicillin; Tc, tetracycline; Ef, enrofloxacin; Nm, neomycin; Col, colistin

**Table 4:** Associations between resistance in *Escherichia coli* and proportion of sows receiving post-farrowing antibiotic treatment

		Proportion of sows receiving post-farrowing antibiotic treatment			
		**< 10%**	**10–19%**	**20–30%**	> 30%
**Trimethoprim-sulphonamide resistance**		12.2%	35.1%	42.9%	44.4%
**Number of herds**		25	37	16	5
**Number of isolates tested**		41	57	28	9

Proportion of herds with *E. coli* resistant to trimethoprim-sulphonamide in relation to proportion of sows receiving post-farrowing antibiotic treatments. Data for 135 isolates from the 83 herds which submitted questionnaires on farm use of antibiotics. Higher proportion of post-farrowing treatments were associated with higher occurrence of antimicrobial resistance in *E. coli* from piglets with diarrhoea

### Comparison of AMR in E. Coli isolates from study samples and from clinical submissions

In the collection period of the study samples, 57 *E. coli* isolates from clinical submissions were susceptibility tested at SVA. Twenty-three of these were from suckling, 30 from weaned piglets, and in four cases the age was unknown. Of these, 44 isolates were tested for enterotoxin genes and 77% were positive. When comparing resistance in isolates from clinical submissions to those from study samples, there was no significant difference in proportion of isolates with resistance to any of the antibiotics tested (*P* > 0.05) (Table 2). The proportion of isolates that were susceptible to all tested antibiotics was 46% (26/57) among clinical submissions and not significantly different from the proportion among study samples (41%, 65/158) (*P* > 0.05).

### Statistical analysis of risk factors for AMR in study samples

Univariable analyses, showed statistical associations (*P* ≤ 0.05) between the estimated proportion of sows being treated with antibiotics post-farrowing and resistance to trimethoprim-sulphonamide, streptomycin, and tetracycline, respectively. The probability of an isolate being resistant to trimethoprim-sulphonamide increased with an increasing proportion of sows receiving post-farrowing antibiotic treatments. There was also a significant association (*P* ≤ 0.05) between age category and resistance to trimethoprim-sulphonamide, with trimethoprim-sulphonamide resistance being more frequent in isolates from suckling piglets (44%; 38/86) as compared to isolates from weaned piglets (29%; 20/70). There was no significant association between a sample originating from a herd with high dose zinc oxide supplementation and any of the resistance phenotypes (*P* > 0.05) (Table 5).

**Table 5:** Occurrence of antimicrobial resistance in *E. Coli* in relation to high dose zinc oxide supplementation

	High dose zinc oxide in feed			
	Never	> 1 year ago	Ongoing	Overall
Number of herds	31	16	36	83
Number of isolates	50	26	59	135
Proportion of isolates with resistance to:				
*Ampicillin*	24%	42%	27%	29%
*Streptomycin*	32%	46%	39%	38%
*Tetracycline*	10%	31%	19%	18%
*Trimethoprim-sulphonamide*	26%	42%	29%	30%

Proportion of *E*. *coli* isolates resistant to ampicillin, streptomycin, tetracycline, and trimethoprim-sulphonamide from pigs in herds that: never; >1 year ago; or ongoing were using zinc oxide. No significant association between AMR and use of hight dose zinc oxide supplementation was observed (*P* > 0.05)

The multivariable regression analysis included 134 samples (i.e., all complete observations) (Table 6). No significant variation was explained by the random variable herd, which means that there was no clustering of the outcome (i.e., trimethoprim-sulphonamide resistance) at herd level. The proportion of sows receiving post-farrowing antibiotic treatment and the age category of piglets from which the sample was collected, were the two fixed variables with a significant association to trimethoprim-sulphonamide resistance in the multivariable model and were consequently kept in the final model. The odds of an *E. coli* isolate to be resistant to trimethoprim-sulphonamide was almost four times as high (OR = 3.6 95% CI 1.2–10.8) in a sample originating from a herd where 10–19% of the sows were treated with antibiotics post-farrowing, as compared to an isolate originating from a herd with less than 10% of the sows being treated post-farrowing. The odds of an isolate to be trimethoprim-sulphonamide resistant from a herd with 20–29% or > 30% of sows treated with antimicrobials post-farrowing were almost six times as high (OR = 5.8, 95% CI 1.7–20.0 and OR = 5.9, 95% CI 1.1–31.0) as compared to a sample originating from a herd with less than 10% of the sows receiving post-farrowing treatment. An isolate originating from neonatal diarrhoea had three times the odds to be trimethoprim-sulphonamide resistant, as compared to an isolate from a post-weaning diarrhoea sample.

**Table 6:** Associations between antimicrobial resistance in *Escherichia coli* from study samples and potential risk factors

Random effect variable	Variance			
Herd	< 0.0001			
**Fixed effect variables**	**Category**	**OR**	**95% CI**	**P > z**
Estimated proportion of sows receiving antibiotic treatment within the first week after farrowing	*< 10%*	*Ref*		
	*10–19%*	3.6	1.2–10.8	0.023
	*20–29%*	5.8	1.7–20.0	0.005
	≥ *30%*	5.9	1.1–31.0	0.035
Type of diarrhoea	*Post-weaning diarrhoea*	*Ref*		
	*Neonatal diarrhoea*	2.7	1.2–6.1	0.019

Results from multivariable mixed logistic regression analysis between potential risk factors and trimethoprim-sulphonamide resistance in *Escherichia coli* isolates from study samples (*n* = 134). Variables without significant association to antimicrobial resistance are not included

**Fig. 1:**
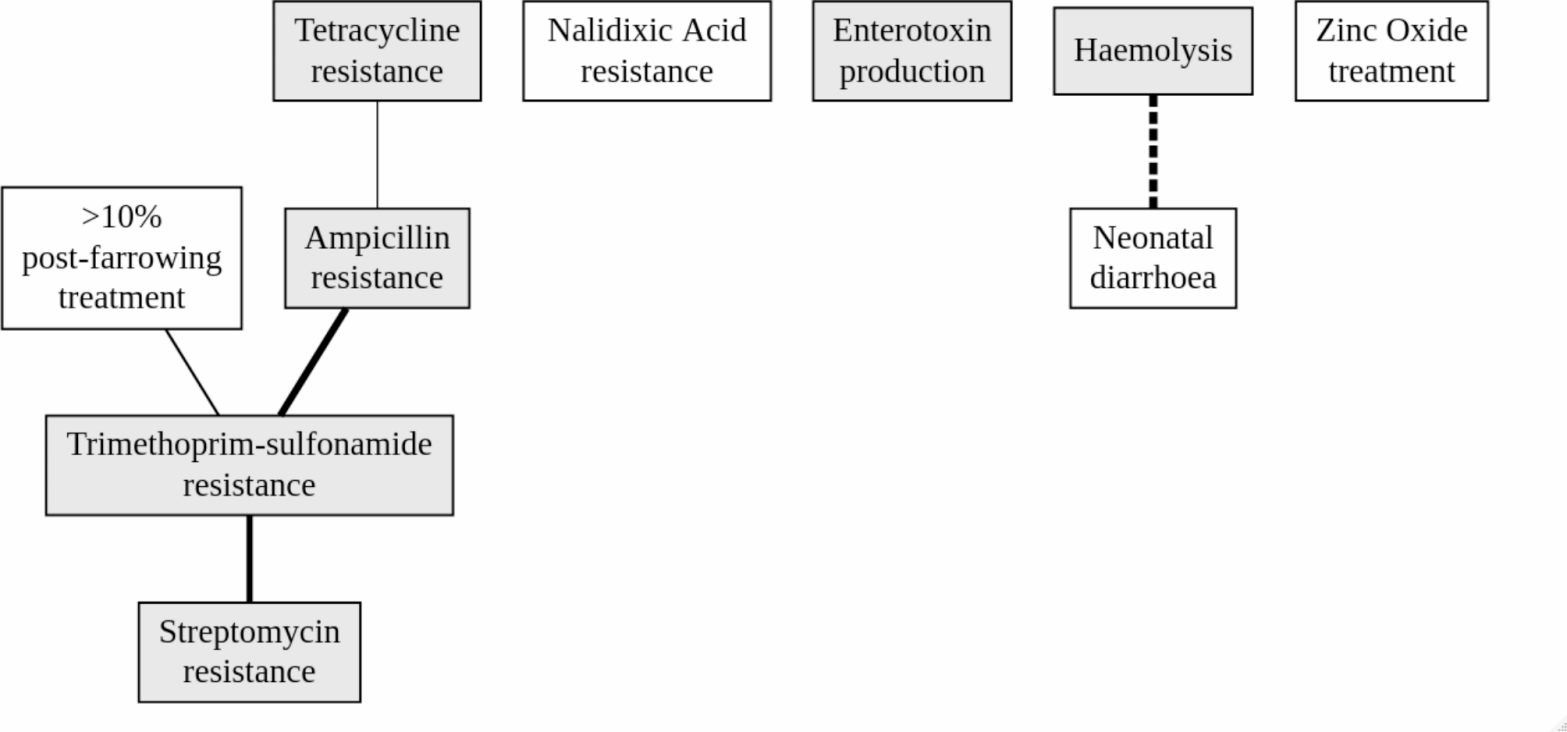
Associations between antimicrobial resistance in *Escherichia coli* from piglets with diarrhoea and potential risk factors. **Legend**. The figure shows graphic results from Additive Bayesian Network (ABN) analysis, a multivariate analysis to investigate associations between all variables. All included variables were binary variables. White squares are variables related to herd or sample and grey squares are variables describing properties of the isolates. Lines between variables indicate direct associations between these variables and the thickness of the line is proportional to the strength of the association. A solid line indicates a positive association e.g., trimethoprim-sulphonamide resistance in an isolate is associated with a higher probability of ampicillin resistance in isolates. The dashed line indicates a negative association, in this case because haemolysis did not occur in any isolates from neonatal diarrhoea.

The DAG resulting from the ABN modelling is shown in Fig. 1, odds ratios and strengths of associations are shown in Table 7. The analysis included 134 complete observations. Tetracycline resistance was weakly directly associated (link strength = 7%) (Table 7) to ampicillin resistance, as indicated by a solid line connecting the two variables (Fig. 1). The associated odds ratio (OR_ampicillin|tretracycline_=4.9) indicated that when the *E. coli* isolate was resistant to tetracycline, it was almost five times more likely to be resistant also to ampicillin (Table 7). Ampicillin resistance in turn, was strongly directly associated (link strength = 30%) to trimethoprim-sulphonamide resistance (OR = 27.2), which in turn was directly associated with streptomycin resistance (OR = 18.4). Trimethoprim-sulphonamide resistance was also associated with the proportion of sows receiving post-farrowing treatment. In herds with more than 10% of the sows receiving post-farrowing antimicrobial treatment, the *E. coli* isolates were 10 times more likely to be resistant to trimethoprim-sulphonamide (OR = 10). Haemolysis of an isolate did not occur in any of the samples from neonatal diarrhoea, resulting in a negative direct association between these two variables.

**Table 7:** Associations between antimicrobial resistance in *Escherichia coli* from study samples and potential risk factors

Link	OR	95% CI	Link strength (%)
Ampicillin resistance| Tetracycline resistance	4.9	2.0–12.9	7
TMP-Sulfa ^a^ resistance| Ampicillin resistance	27.2	9.5–93.4	30
TMP-Sulfa resistance| >10% post-farrowing treatment	10	2.8–45.1	11
Streptomycin resistance| TMP-Sulfa resistance	18.4	7.5–50.1	26
Neonatal diarrhoea| Haemolysis	NA ^b^	NA	35

Results from a multivariate analysis, Additive Bayesian Network (ABN) analysis investigating associations between all variables on which data were collected in this study. Results on odds ratios (OR), 95% confidence intervals (CI) and link strength of significant associations between variables are shown. ^a^ TMP-Sulfa = trimethoprim-sulphonamide, ^b^ Estimate not available due to complete data separation

The remaining variables included in the model, i.e., high dose zinc oxide supplementation in the feed, nalidixic acid resistance and presence of enterotoxin genes in the isolate, showed no associations with any of the other variables.

## Discussion

The main aim of this study was to investigate if occurrence of AMR in *E. coli* cultivated from clinical submissions of intestinal content from piglets with diarrhoea, on which the Swedish national monitoring program is based, could be biased and overestimate occurrence of AMR in isolates from the general population of piglets with diarrhoea. The risks of such overestimations and the need to validate data based on clinical submissions was emphasised in building the European Antimicrobial Resistance Surveillance network in veterinary medicine (EARS-Vet) [23]. However, in the present study, we found no difference in occurrence of AMR in *E. col*i from study samples and from concurrent clinical submissions to SVA. This agrees with results of a similar, but smaller, Swedish study conducted in 2020 [4] and indicates that, in Sweden, AMR in *E. coli* from clinical submissions to SVA provide a good estimate of the general situation in diarrhoeic piglets in the country.

The study also shows that resistance to trimethoprim-sulphonamide, streptomycin, ampicillin, and tetracycline is common in *E. coli* from piglets with neonatal and post-weaning diarrhoea and that many isolates are co-resistant to two or more of these antibiotics. Resistance to enrofloxacin, neomycin and colistin was less common and nitrofurantoin and cefotaxime resistance was not observed. Common occurrence of resistance limits therapeutic choices for piglet diarrhoea and warrants regular susceptibility testing of isolates to guide selection of therapy in a herd.

On selective culture, quinolone resistant *E. coli* (QREC) was found in about one-third of the samples and did not differ markedly between suckling and weaned piglets. This contrasts with a recent Swiss study where occurrence of QREC on farms using fluoroquinolones was about 90% in suckling piglets and about 10% in weaners [24]. In the Swiss study, QREC was found also on farms not using fluoroquinolones, albeit at lower levels, and the authors conclude that apart from restricted use of fluoroquinolones, measures to hinder spread of QREC are needed to reduce occurrence of quinolone/fluoroquinolone resistance. In our study, presence of QREC was not associated with any of the potential risk factors evaluated, but as use of fluoroquinolones in the herds was not documented in detail, possible associations were not completely evaluated.

This study was conducted in 2016–2017, but similar levels of AMR and multiresistance in *E. coli* from pigs as observed here were reported from Svarm in the period thereafter [4]. However, since 2019 a decreasing trend in resistance to trimethoprim-sulphonamide and ampicillin, as well as in multiresistance, is reported in Svarm [4].

A second aim of this study was to investigate potential risk factors for AMR in *E. coli* from Swedish piglets with diarrhoea, part of a livestock population with one of Europe´s lowest sales of antibiotics for food producing animals [5]. Moreover, antibiotic use in Swedish farrow-to-finish herds is lower than in similar herds in other European countries [25]. However, the present study showed that some herds frequently treat a large proportion of sows post-farrowing. The first-choice antibiotic used for these treatments was trimethoprim-sulphonamide in most (70%) of the herds, a choice that agrees with the current guidelines for the use of antibiotics in production animals published by the Swedish Veterinary Association [26]. The proportion of sows treated post-farrowing was directly associated with resistance to trimethoprim-sulphonamide in *E. coli* from diarrhoeic piglets. Thus, isolates from herds with more post-farrowing treatments were more likely to be resistant than isolates from herds with less treatments. This is logical as trimethoprim-sulphonamide was the most frequent first-choice antibiotic and agrees with previous work from Belgium which identified antibiotic administration to the sow as a risk factor for resistant *E. coli* in the piglet [27]. Also, in a longitudinal study from Germany were *E. coli* from piglets more likely to be resistant to ampicillin if the sow carried ampicillin-resistant isolates [28]. In addition, the present study showed that the probability of resistance to trimethoprim-sulphonamide was markedly higher in herds treating 10–19% of the sows post-farrowing, as compared to herds treating < 10% of sows post-farrowing. The difference was less pronounced between herds treating 10–19% and herds treating > 20% of the sows. This suggests that even a relatively low antibiotic usage drives resistance and therefore it is probably necessary to reduce antibiotic treatments substantially to achieve low levels of AMR as previously suggested [24]. This study does not point to any thresholds for antibiotic use in relation to AMR which is, at least partly, because it was not designed to address this question. The variable describing the level of antibiotic use (proportion of sows receiving post-farrowing treatment) was based on farmers’ estimates and not on the amounts and active substances used. The estimated use had to be categorised as the farmers’ estimates were not continuous, we interpreted this as farmers being likely to round off estimates to the nearest tenth, impeding identification of a threshold.

The ABN modelling showed that, in addition to the above-mentioned direct association, there were also indirect associations between the level of post-farrowing antibiotic treatments and resistance to streptomycin, tetracycline and ampicillin. This suggests that the use of a single antibiotic, in this case trimethoprim-sulphonamide, not only has impact on resistance to this substance, but it can also drive emergence of resistance to other antibiotics, in this case streptomycin, ampicillin and tetracycline. There was also frequent occurrence of multi-resistant isolates, with the most common phenotype being resistance to ampicillin, streptomycin and trimethoprim-sulphonamide. Similar associations were shown in an Irish study [29] and is presumably explained by co-selection [30].

In this study, isolates from suckling piglets with diarrhoea were more likely to be resistant than isolates from weaned piglets. It was previously shown that *E. coli* from neonatal piglets were more likely to be resistant to ampicillin and azithromycin if *E. coli* isolates from the sow were resistant to these antibiotics [28]. Also, an association between quinolone treatment of sows and quinolone resistance in *E. coli* from piglets have been found, although the proportion of resistant isolates had decreased when the piglets had reached an age of two months [31]. Similar observations for quinolone resistance were made also by others [24]. This agrees with our results and suggests that, given that the piglets are not treated with antibiotics, resistance in gastrointestinal *E. coli* will decrease over time.

The use of zinc oxide to prevent diarrhoea in weaned piglets has been common in Swedish herds. However, since the ban on the use of medicinal zinc oxide came into force in June 2021 in the EU, high dosed zinc oxide is no longer used to prevent post-weaning diarrhoea. The proportion of herds that used high dose zinc oxide (41%) in the present study was similar to what was found in a Swedish study of 60 farrow-to-finish herds conducted three years earlier [8]. Zinc oxide treatment has been shown to be associated with AMR [9, 32] but in the present study, no such association was seen. However, the number of observations was limited, and a weaker association could still be present.

There are some aspects of the present study that could have impact on the representativeness of the results. Selection of farms was by convenience and not random, and it is possible that farmers experiencing treatment failures were more likely to participate. If so, AMR occurrence may be overestimated. On the other hand, it is also possible that farmers with a greater concern and interest in these issues participated. It has been shown that among farmers, a higher perceived risk on the use of antibiotics was related to lower usage on the farm [33]. Thus, the farms participating in our study could be farms with lower antibiotic use than average and consequently, AMR occurrence could be underestimated. Furthermore, a previous Danish study showed that AMR was more common in enterotoxin producing *E. coli* from weaned piglets than in non-enterotoxin producing isolates [34]. This means that occurrence of AMR could be underestimated in the present study as enterotoxin producing *E. coli* were less common than expected. However, occurrence of AMR in isolates from the study samples was similar to that in isolates from clinical submissions with a high proportion of enterotoxin producing isolates. Moreover, the trend of increasing resistance to trimethoprim-sulphonamide and ampicillin in clinical isolates *of E. coli* during the study period is similar in indicator *E. coli* from healthy fattening pigs, which supports the validity of results from the present study [6].

## Conclusions

Clinical submissions can be used to monitor AMR of *E. coli* from pigs with diarrhoea in Sweden, as no indications of overestimation of AMR occurrence in clinical submissions were seen in this study. High post-farrowing treatment rates in sows were associated with a higher probability of AMR in *E. coli* from suckling and post-weaning pigs with diarrhoea, also at low treatment rates. This was seen especially for trimethoprim-sulphonamide resistance, the most used antibiotic, but also for other antibiotics most likely through co-selection. These findings indicate that antibiotic use needs to be reduced substantially to achieve a reduction of AMR. No association between AMR and use of high dose zinc oxide to weaned piglets was found.

## Data Availability

The datasets used and analysed during the current study are available from the corresponding author on reasonable request.

## Abbreviations

AMR: Antimicrobial resistance
ABN: Additive Bayesian Network
DAG: Directed acyclic graph
ETEC: Enterotoxigenic *E. coli*
EUCAST: European committee on antimicrobial susceptibility testing
PCR: Polymerase chain reaction
TMP-Sulfa: Trimethoprim-sulphonamide
QREC: Quinolone resistant *Escherichia coli*
